# Retraction: ZASP Interacts with the Mechanosensing Protein Ankrd2 and p53 in the Signalling Network of Striated Muscle

**DOI:** 10.1371/journal.pone.0298249

**Published:** 2024-01-31

**Authors:** The PLOS ONE Editors

After this article [1] was published, concerns were raised about results reported in Figs 2, 5, and 6.

Specifically:

- Lanes 2 and 3 of the IP anti-Flag Wb: anti-Ankrd2 panel in Fig 2C appear similar to lanes 5 and 6 of the IP anti-Flag Wb: anti-p53 in Fig 5B.
- In Fig 2D, when levels are adjusted to visualize background, the 4^th^ and 5^th^ (“GST” and “-”) Wb: anti-GST panels appear similar.
- In the left Wb: anti-FLAG Panel in Fig 5B, when levels are adjusted to visualize background there are several areas that appear to contain repeating or similar patterns.
- In Fig 5C the 4^th^ and 5^th^ (“GST” and “-”) Wb: anti-GST panels appear similar when the latter is horizontally stretched. The bands in these lanes also appear similar to those in the third WB: anti-His panel in Fig 6D.
- In Fig 5C, all five Wb: anti-His panels appear similar.

The corresponding author stated that the original data underlying results in this article are no longer available.

In light of the unresolved concerns discussed above, the *PLOS ONE* Editors retract this article.

GF did not agree with the retraction. VCM, WBK, SK, NV, ZL, AB, PM, LB, MV, and GV either could not be reached or did not respond directly.
